# Segmentation of Planar Surfaces from Laser Scanning Data Using the Magnitude of Normal Position Vector for Adaptive Neighborhoods

**DOI:** 10.3390/s16020140

**Published:** 2016-01-22

**Authors:** Changjae Kim, Ayman Habib, Muwook Pyeon, Goo-rak Kwon, Jaehoon Jung, Joon Heo

**Affiliations:** 1 Department of Civil and Environmental Engineering, College of Engineering, Myongji University, 116 Myongji-ro, Cheoin-gu, Yongin, Gyeonggy-do 449-728, Korea; cjkim@mju.ac.kr; 2 School of Civil Engineering, Purdue University, 550 Stadium Mall Dr, West Lafayette, IN 47907, USA; ahabib@purdue.edu; 3 Department of Civil Engineering, Konkuk University, Neungdong-ro, Gwangjin-gu, Seoul 143-701, Korea; neptune@konkuk.ac.kr; 4 Department of Information and Communication Engineering, Chosun University, 309 Pilmundae-ro, Dong-gu, Gwangju 501-759, Korea; grkwon@Chosun.ac.kr; 5 Department of Photogrammetry, University of Bonn, Nussallee 15, 53115 Bonn, Germany; Jhjung1215@gmail.com; 6 School of Civil and Environmental Engineering, College of Engineering, Yonsei University, 50 Yonsei-ro, Seodaemun-gu, Seoul 120-749, Korea

**Keywords:** laser scanning data, normal position vector, segmentation, planar patches, attribute homogeneity

## Abstract

Diverse approaches to laser point segmentation have been proposed since the emergence of the laser scanning system. Most of these segmentation techniques, however, suffer from limitations such as sensitivity to the choice of seed points, lack of consideration of the spatial relationships among points, and inefficient performance. In an effort to overcome these drawbacks, this paper proposes a segmentation methodology that: (1) reduces the dimensions of the attribute space; (2) considers the attribute similarity and the proximity of the laser point simultaneously; and (3) works well with both airborne and terrestrial laser scanning data. A neighborhood definition based on the shape of the surface increases the homogeneity of the laser point attributes. The magnitude of the normal position vector is used as an attribute for reducing the dimension of the accumulator array. The experimental results demonstrate, through both qualitative and quantitative evaluations, the outcomes’ high level of reliability. The proposed segmentation algorithm provided 96.89% overall correctness, 95.84% completeness, a 0.25 m overall mean value of centroid difference, and less than 1° of angle difference. The performance of the proposed approach was also verified with a large dataset and compared with other approaches. Additionally, the evaluation of the sensitivity of the thresholds was carried out. In summary, this paper proposes a robust and efficient segmentation methodology for abstraction of an enormous number of laser points into plane information.

## 1. Introduction

There has been significant interest in developing segmentation methodologies useful for the various applications such as Digital Building Model creation, large-scale ortho-photo generation, city modeling, object recognition, and photorealistic 3D modeling. Since the importance of measurements from laser scanning systems was recognized, diverse segmentation approaches have been suggested and proposed to make their processes more efficient, robust, and automated. Laser point segmentation techniques can be roughly divided into two categories [1].

The first category includes the methodologies that segment point clouds based on criteria such as point proximity and attribute similarity, both locally estimated from the surface. For example, scan line segmentation [2,3], and the surface growing method [4] belong to this first category. Briefly, the scan line segmentation method first splits each scan line into straight line segments and then, based on a similarity criterion, merges them with adjacent scan line segments. The split-and-merge approach to image segmentation is applied to 3D space. On the other hand, the surface growing method extends seed regions to adjacent points by considering the proximity and similarity of points [4,5,6,7,8]. In other words, the points that are located near the seed region and that have attributes similar to those of the seed region are categorized into the same group as is the seed region. Also, RANSAC (RANdom SAmple Consensus) [9] is a well-known method to generate a number of model proposals by randomly sampling data points in the object space and then select the model with the largest set of inliers. However, this method is designed to extract a single model. Other alternatives such as Sequential RANSAC and MultiRANSAC algorithms are proposed for multi-model fitting [10,11,12]. Additionally, RHA (Residual Histogram Analysis) implements preference analysis which inspects the distribution of residuals of individual data point with respect to the models [13]. Similarly, J-linkage is suggested to avoid a consensus oriented approach by working in the preference space. It adopts a conceptual representation of points. Points belonging to the same structure will have similar conceptual representations [14,15].

Methods belonging to the second category of laser point segmentation techniques aggregate points with similar attributes into clusters in an attribute space (or parameter space), not, as with the scan line segmentation and surface growing methods just discussed, in the object space. The well-known Hough transform [16] can be extended to deal with 3D surfaces [17]. One laser point on a planar surface in the object space defines a plane in the attribute space. Therefore, points on the same plane intersect in the attribute space at the position that corresponds to the slope and distance of the plane. Within the implementation procedure, the attribute space is quantized and the points voted into the accumulator array cell with the highest number of planes, which represent the largest number of points on the corresponding planar surface in the object space, can be identified and segmented. There is also a publication about applying Hough transform to formulate multi-model fitting as a clustering problem and use mean-shift [18] to identify the modes in this Hough space [19]. Another methodology performs clustering based on an attribute vector consisting of the surface texture measures and/or the parameters of the tangent plane defined from the points in the neighborhood of a certain point [20]. Several variations and applications have been accomplished by [21,22,23,24]. Hofmann [21] suggested that rather than using an attribute vector with seven parameters [20], a clustering approach, based on an attribute vector with three parameters for each triangle of a Triangulated Irregular Network (TIN)-structure, should be followed. Filin and Pfeifer [22,23] implemented normal-vector-based clustering using a slope adaptive neighborhood. To reduce the dimensions of the attribute space for greater efficiency or memory requirement purposes, they first carried out the clustering process in a two-dimensional attribute space, utilizing two normal vector slopes in the x and y directions. Afterwards, all of the points with the same slope but different z-intercepts were clustered in the one-dimensional attribute space. Biosca and Lerma [24] employed an attribute-based clustering approach to the segmentation of planar surfaces. Specifically, they presented an unsupervised planar segmentation process based on fuzzy clustering. Yang and Dong [25] and other two studies [26,27] suggested similar shape-based segmentation methods for laser scanning point clouds. They proposed methods to calculate the optimal neighborhood size based on the eigenvalues and entropy function, to derive the geometric features for point classification, and to segment the classified point clouds according to the principle of region growing.

In our view, most of the above-mentioned segmentation techniques suffer from the following problems: (1) Surface-growth-based segmentation depends heavily on seed point (or seed region) selection, and segmentation performance can be sensitive to the choice of seed points [28]. RANSAC based methods and J-linkage takes greedy clustering approaches which explicitly or implicitly maximize the number of inliers. In these cases, there is possibility that some random model can have a larger number of inlier than the true models [19]; (2) The attribute-space-based segmentation techniques, especially those based on Hough transform, do not consider the connectivity of points, which fact causes the segmentation results to contain many spurious surfaces that do not actually exist [17,20]; (3) The attribute-vector-based clustering techniques do not involve seed points; however, the efficiency issue arises. Notably, the dimensions of the memory space should be the same as the number of parameters that the utilized attribute vector has. In this regard, with an increased number of attributes utilized in the clustering procedure, the amount of memory required also increases, and tremendously. For example, in the case of [20], a seven-dimensional memory space is required, since his clustering approach is based on a seven-parameter attribute vector; (4) Most of the previous studies pertaining to segmentation techniques have focused on utilization of only one type of dataset (*i.e.*, either airborne or terrestrial laser scanning data). Considering the data integration trend for various 3D applications, a generic segmentation approach is essential in order to deal with both the airborne and terrestrial domains.

In this regard, the specific objectives of the present research are to propose a segmentation approach that (1) considers both the similarity (in the attribute space) and the proximity (in the object space) of laser points simultaneously; this will remove the problems from the above-mentioned approaches (*i.e.*, seed point selection problem, and clustering problem due to greedy inlier maximization); (2) reduces the dimensions of the attribute space for efficiency or memory requirement purposes; and (3) works well with both airborne and terrestrial laser scanner point clouds.

This paper commences with a description of the methodology proposed for laser point segmentation. Neighborhood definition, attribute computation based on the defined neighborhood, and the procedures for points clustering using the derived attributes are addressed. Next, test datasets acquired from airborne and terrestrial laser systems are described, after which segmentation experiments carried out in the present study are discussed based on the qualitative and quantitative evaluations. Additionally, the evaluation of the sensitivity of the thresholds was carried out. Finally, in concluding remarks, findings are summarized and future work is recommended.

## 2. Proposed Segmentation Methodology

The segmentation procedure proposed in the present study includes three steps: (1) neighborhood definition; (2) attribute computation; (3) clustering of points. One should note that the proposed methodology focuses on planar segmentation since most of the man-made structures consist of planar patches. As the first step, the neighborhood definition adopted in this study considers both the three-dimensional proximity between points and the shapes of surfaces, in order to increase the homogeneity among neighbors. Point attributes, then, are computed using the neighboring points identified as based on the neighborhood definition.

Subsequently, an attribute vector with two parameters is acquired and utilized to reduce the dimensions of the attribute space. Afterwards, clustering of points on the same plane is carried out while considering, simultaneously, the similarity in the attribute space and the proximity in the object space. These three segmentation-procedural steps will be explained in detail in the following subsections.

### 2.1. Adaptive Cylindrical Neighborhood Definition

**Figure 1:**
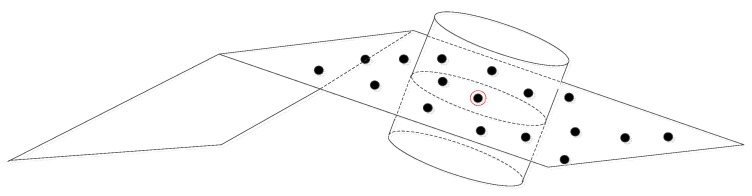
Schematic concept of the adaptive cylinder neighborhood definition.

The way in which the neighborhood of a laser point is defined significantly affects the set of attributes computed for that point. In this research, a definition that considers both the three-dimensional relationships between irregularly-distributed points and the shapes of surfaces (especially, planes) is applied. The shapes of the surfaces on which associated points are located are incorporated into the neighborhood definition. This means that the adopted neighborhood definition considers, in taking into account the proximity of the points, only those points that are located on the same surface. Points on different surfaces, on the other hand, are not considered to be neighbors, even if they are spatially close. This definition increases the homogeneity among neighbors. Conceptually, the neighborhood definition starts by determining a cylinder whose axis is normal to the surface to which the point of interest belongs (refer to Figure 1). Significantly, the axis of this cylinder will be changed according to the orientation of the surfaces. It is for this reason that this neighborhood definition is referred to as the adaptive cylindrical neighborhood definition. The present research introduced an iterative procedure and considered the weights of points in order to make the neighborhood definition more reliable.

At this stage, one should note that it is not necessary to directly determine the axis of the cylinder in this study. In other words, the cylinder will be determined from the plane fitting and a buffer threshold. More detailed explanation of the neighborhood definition procedure will be as follows.

(1) Define the neighborhood by a sphere that is centered on the considered point.
(2) Compute an initial fitted plane using the neighboring points determined in step 1.
(3) Calculate the shortest Euclidean distances between the plane computed in step 2 and the associated points belonging to the neighborhood.
(4) Construct a weight matrix while considering values of the distances calculated in step 3.
(5) Re-compute a plane using the weight matrix and the neighboring points.
(6) Repeat steps 3 to 5 until plane parameter convergence or the completion of a maximum number of iterations.
(7) Determine the final neighborhood after discarding points (*i.e.*, outliers) whose distances are higher than a determined threshold.
(8) Apply steps 1 to 7 for every point in the dataset

**Figure 2:**
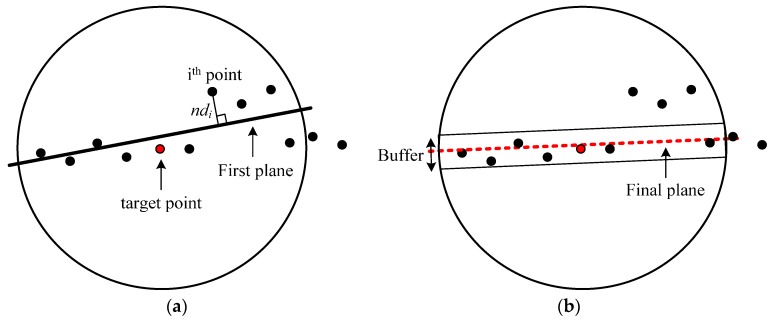
(**a**) The first fitted plane for a spherical neighborhood centered at a point in question; and (**b**) the final fitted plane derived through the iterative procedure using the weight function (side view).

More specifically, the neighborhood definition starts by establishing a spherical neighborhood centered at each point (this neighborhood definition appears in [29]). In other words, the radius of the sphere centered on the considered point is determined to include a sufficient number of points to define reliable attributes, or “plane parameters”. At this stage, we can consider point spacing, possibility of having outliers within those points (*i.e.*, points that do not belong to the planar surface), and noise level within the datasets. The points in this spherical neighborhood are used to determine, through a least squares adjustment procedure, an initial fitted plane for the point in question. Since this research deals with airborne and terrestrial data, the datasets contain various planar patches with different slopes and aspects. An appropriate mathematical representation of such planar patches is, therefore, applied in this research, as seen in Equations (1) and (2):

(1) aX+bY+cZ=d

(2) $$a^{2}+b^{2}+c^{2}=1$$

where (X,Y,Z) are the coordinates of a point, (a,b,c) are the direction cosines of the surface normal vector of the plane, and d is the shortest Euclidean distance between the origin of the coordinate system and the plane. The result of the initial plane fitting using the points belonging to a spherical neighborhood is illustrated in Figure 2a. After the first plane is derived, the shortest Euclidean distances between the plane and the associated points are computed, as illustrated in Figure 2a. The distances are then used as factors when the weights, for the next iteration of the plane fitting procedure, are determined (see Equations (3) and (4)) according to Huber method [30].

(3) $$\begin{array}{l} p_{i}=1\text{ for }nd_{i}\leq \sigma \\ P_{i}=\frac{\sigma }{nd_{i}}\text{ for }nd_{i}>\sigma \end{array}$$

(4) $$nd_{i}=\frac{\left|ax_{i}+by_{i}+cz_{i}-d\right|}{\sqrt{a^{2}+b^{2}+c^{2}}}$$

where $p_{i}$ is the weight of the *i-*th point, $nd_{i}$ is the shortest Euclidean distance between the plane and the *i-*th point, $\left(x_{i},y_{i},z_{i}\right)$ are coordinates of the *i-*th point, and σ is the accuracy of the laser data in the depth direction.

This procedure is repeated until the plane parameters cease to change from one iteration to the next or until the maximum number of iterations (e.g., 10 iterations) is reached. Afterwards, a buffer is defined above and below the final fitted plane. The size of this buffer depends on the noise level expected in the laser datasets. More practically, two times the accuracy of the laser data in the depth direction (covering around 95% of the laser points on the plane) is set for the above and below parts of the final plane. Finally, the neighborhood is defined by the points located inside the established cylinder (see Figure 2b). The points whose distances (*i.e.*, $nd_{i}$ s) are less than the determined threshold are selected as the members of the neighborhood; otherwise they are rejected as shown in the following formulas:

(5) $$\begin{array}{l} SN=\{s_{1},s_{2},\ldots ,s_{n}\}\\ s_{i}\in CN\;if\;nd_{i}\leq threshold\\ s_{i}\notin CN\;if\;nd_{i}>threshold \end{array}$$

where *SN* is the set of points belonging to the spherical neighborhood, $s_{i}$ is the *i-*th point in the neighborhood, *CN* is the set of points belonging to the adaptive cylindrical neighborhood, and *threshold* is 2σ as above-mentioned.

Moreover, the point of interest is not considered in the remaining procedures of this study if the iteration stops after the completion of a maximum number of iterations. One should note that the points that are already involved in the neighborhood for a certain target point are not excluded in the procedure of neighborhood definition for another target point.

### 2.2. Attribute Computation Using the Neighboring Points

**Figure 3:**
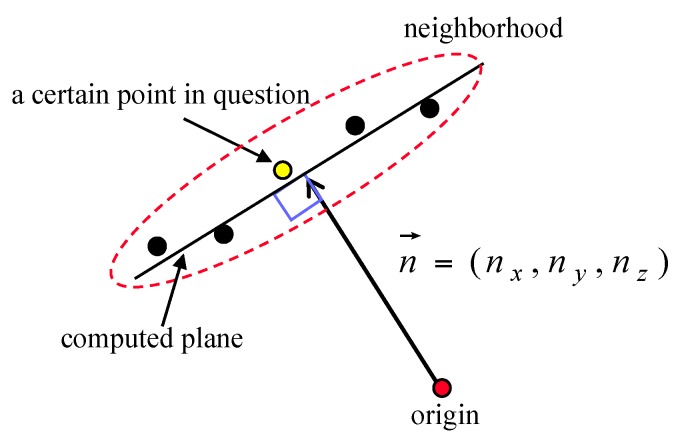
Vector defined from the origin to the computed plane for a given point, using its neighboring points.

In this research, point attributes were computed based on the neighboring points identified using the defined neighborhood. After introducing an origin, a normal vector from the origin to the plane for a certain point in question was defined as seen in Figure 3. Since this normal vector extends from the reference point (*i.e.*, the origin introduced) to the specific position at which the plane and the vector orthogonally intersect with each other, the vector can be also called a position vector. To differentiate the vector seen in Figure 3 from the conventional definitions of a normal vector and a position vector, it is referred to as *a normal position vector* in this research.

Most techniques based on attribute clustering use a voting scheme with an accumulator array that is constructed in the attribute space. The dimensions of the accumulator array depend on the number of attributes utilized by the technique in question. The three components of the normal position vector can be used as attributes in the voting scheme. It should be noted that one normal position vector defines one plane without any mathematical ambiguity. In this regard, the segmentation technique that uses the three components of the normal position vector partitions points into distinct regions without any segmentation ambiguity, excepting that arising from co-planarity (*i.e.*, planes sharing the same normal position vector but disconnected in the object space). However, use of these attributes requires the construction of an accumulator array that has three dimensions, one for each vector component. Unfortunately, a voting scheme that uses a three-dimensional accumulator array is computationally expensive. To reduce the three-dimensional accumulator array to two dimensions, the slopes of the normal vector in the x and y directions were used as attributes for the planar patch segmentation, rather than using all three normal vector components to handle airborne laser scanning data [22,23,31]. Even though this method reduces the dimensions of the accumulator array, segmentation ambiguity remains a problem. If this method is used, parallel planes that have the same normal vector slopes in the x and y directions but different offsets in the z direction are segmented as one group. This problem was resolved, after completion of segmentation in the two-dimensional attribute space, through an additional process; that is to say, by either spatial analysis of the data or the introduction of a one-dimensional accumulator array for the offsets in the z direction.

**Figure 4:**
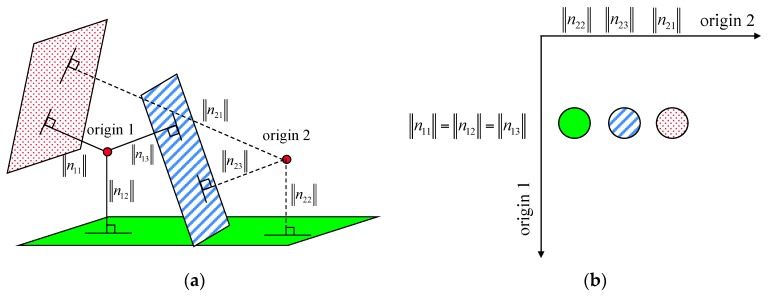
(**a**) Situation in which one origin is located at an equal distance from the planes and the other origin is not; and (**b**) the recorded votes in a two-dimensional accumulator array.

In this research, the magnitude of the normal position vector, $\left||\vec{n}|\right|$, was utilized as an attribute. The magnitude is also same as the shortest Euclidean distance between the origin and the defined plane. One origin can be used for planar patch attribute computation. However, different planes with the same magnitudes of normal position vectors can exist by chance. Planes tangential to a sphere centered on the origin are examples of this case. The points belonging to these planes will have the same attribute values. Therefore, two origins were introduced in the present research; significantly, this reduces the possibility of the occurrence of the ambiguity whereby points belonging to planes with different slopes and aspects will have the same attribute values. Figure 4a illustrates the situation in which one origin is located at an equal distance from the planes and the other origin is not. If one origin (origin 1) is located at an equal distance from the planes (*i.e.*, $\left||n_{11}||=||n_{12}||=||n_{13}|\right|$), the points belonging to these planes will have the same attribute values. Therefore, it would be impossible to separate these points into different groups in the attribute space. This problem can be resolved by introducing another origin (origin 2). The magnitudes of the normal position vectors from the planes to the second origin will be different from one another (*i.e.*, $\left||n_{21}||\neq ||n_{22}||\neq ||n_{23}|\right|$). Hence, the points belonging to different planes will have different pairs of attributes after two different origins are introduced. These attributes are utilized in the clustering procedure through the voting scheme. It should be noted that the number of origins determines the dimensions of the accumulator array. If there is one origin for planar patch attribute computation, only a one-dimensional accumulator array is necessary for the voting scheme. Since, in the present research, two origins were introduced to reduce the possibility of the above-mentioned ambiguity, a two-dimensional accumulator array was utilized. Figure 4b is a diagram of the accumulator array for the points belonging to the planes in Figure 4a. All of these points are recorded in the accumulator array according to their attributes. As shown in Figure 4b, these points are recorded at the same location along the origin 1-axis. However, they are recorded at different locations along the origin 2-axis, according to the planes to which they belong. The points belonging to the different planes are recorded at the different locations in the two-dimensional accumulator array while avoiding ambiguity. The main advantage of the introduction of two origins while utilizing the magnitudes of the normal position vectors of the points is that the dimensions of the utilized accumulator array are reduced; rather than using 3-dimensional attribute space.

**Figure 5:**
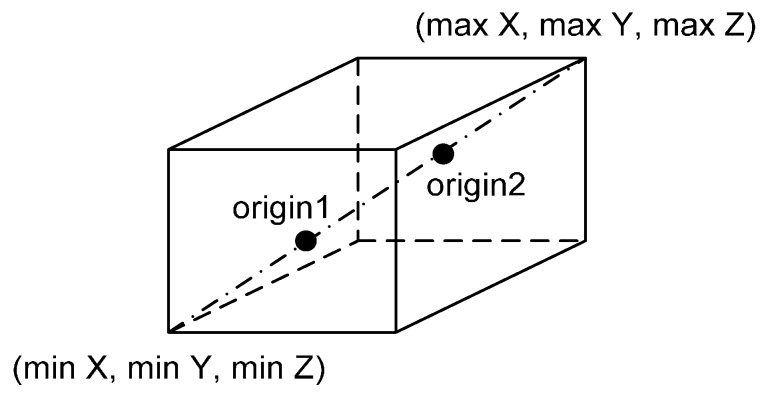
Locations of two origins.

The locations of the two origins are selected according to the way in which these origins are well distributed in the utilized dataset. Once the minimum and maximum values of the laser point cloud in question are recognized, the locations of the two origins are set by Equation (6) (also, refer to Figure 5).

(6) $$origin\;1=\left(\begin{array}{c} min\;X\\ min\;Y\\ min\;Z \end{array}\right)+\frac{1}{3}\left(\begin{array}{c} max\;X-min\;X\\ max\;Y-min\;Y\\ max\;Z-min\;Z \end{array}\right)origin,\;2=\left(\begin{array}{c} min\;X\\ min\;Y\\ min\;Z \end{array}\right)+\frac{2}{3}\left(\begin{array}{c} max\;X-min\;X\\ max\;Y-min\;Y\\ max\;Z-min\;Z \end{array}\right)$$

**Figure 6:**
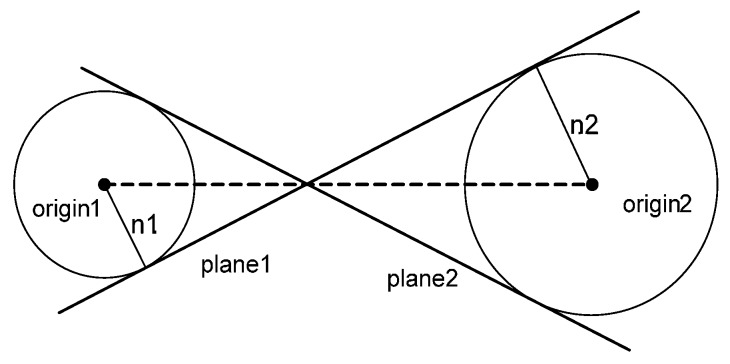
Example of ambiguity from two origins (side view).

By positioning the two origins in this manner, the possibility of segmentation ambiguity is significantly reduced. However, there might still be the chance of ambiguity that causes the attribute vectors of different planes to be located at the same position in the accumulator array; see Figure 6 as an example. The position of plane 1 in the attribute space is (n1, n2); n1 and n2 are the magnitudes of the normal position vectors to plane 1 from the two origins. However, plane 2 shares the same locations in the attribute space. Although the likelihood of this ambiguity occurring in reality is extremely low, the possibility cannot be ignored. In this regard, further processing has to be undertaken to remove the ambiguity thoroughly. This additional processing is designed to detect and resolve the segmentation ambiguity, and is implemented in the clustering procedure. The methodology by which coplanar points are derived using these attributes follows.

### 2.3. Clustering of Points

**Figure 7:**
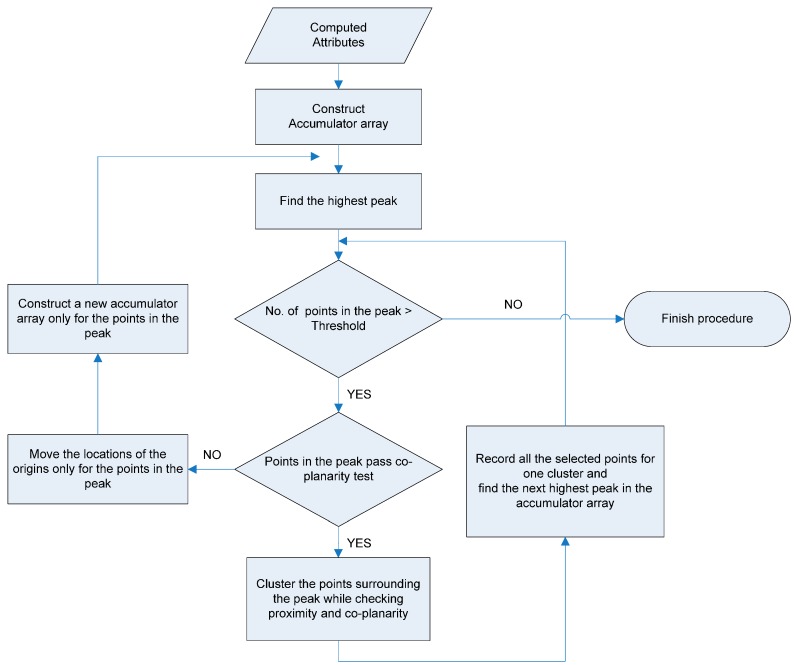
Flow chart of the clustering procedure.

**Figure 8:**
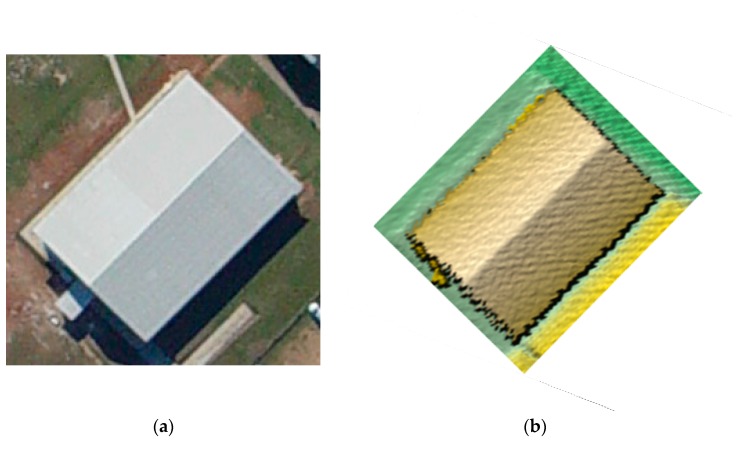

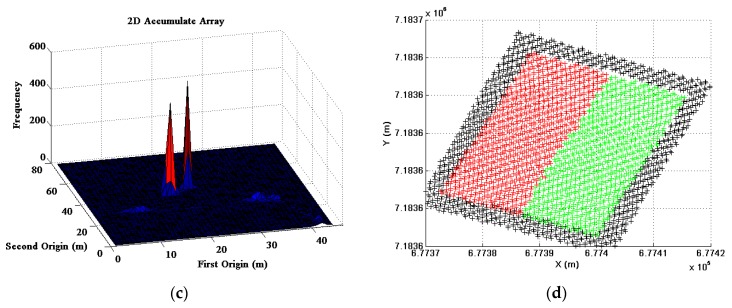
Aggregation of points with similar attributes: (**a**) the digital image; (**b**) the Digital Surface Models over the area of interest; (**c**) the accumulator array; and (**d**) the segmented patches.

Once the locations of the two origins are determined, the attributes of all of the points in the dataset are computed (according to the methods explained in the previous sub-sections) and recorded in the accumulator array. Afterwards, clustering of points with similar attributes is conducted while considering the points’ proximity and co-planarity. Figure 7 is a flow chart of the clustering procedure proposed in this research. The points belonging to different planes in the object space are expected to form different accumulated peaks in the attribute space. Figure 8 illustrates an example of planar patch segmentation. A laser dataset over a region that includes a building with two planar patches (e.g., a gable roof) is selected; see Figure 8a,b. The points that belong to the patches produce, through the voting scheme, two peaks in the accumulator array; see Figure 8c.

Afterwards, the points contributing to the highest peak cell are identified. Here, the cell size of the accumulator array is determined based on the noise level of the laser scanning data utilized (e.g., two times the accuracy of the laser data in the depth direction to cover around 95% of the laser points on the plane). Using these points, plane fitting is conducted through the least squares adjustment procedure. Then, the quality of plane fitting is evaluated by checking the RMS (Root Mean Square) of the shortest Euclidean distances between the points and the plane determined. The acceptable quality of plane fitting is determined while considering the noise level of the laser data utilized. More specifically, the quality of plane fitting will be accepted when the RMS of the distances is less than the accuracy of the laser data. Poor plane fitting will occur when the ambiguity mentioned in the attribute computation procedure exists, though that possibility is extremely low. In other words, the points belonging to the planes with different slopes and aspects might contribute to the highest peak, thus leading to the poor plane fitting result. In this case, the ambiguity is resolved by introducing, only for the points causing the problem, two new origins with different locations. The locations of the new origins are determined by slightly moving the original ones. More specifically, the coordinates of the new origin 1 are computed by adding random values to the coordinates of the original one. The same rule is also applied to the new origin 2 (refer to Equation (7)):

(7) $$\left(\begin{array}{c} X_{new}\\ Y_{new}\\ Z_{new} \end{array}\right)=\left(\begin{array}{c} X_{original}\\ Y_{original}\\ Z_{original} \end{array}\right)+\left(\begin{array}{c} Range\times Rand\_X\\ Range\times Rand\_Y\\ Range\times Rand\_Z \end{array}\right)$$

where, $\left(X_{new},Y_{new},Z_{new}\right)$ are the locations of the new origin, $\left(X_{original},Y_{original},Z_{original}\right)$ are the locations of the original one, Range is a threshold according to which the new origin is located within a certain range (*i.e.*, 5 m), and (Rand_X,Rand_Y,Rand_Z) are the random values between 0 to 1 for the (*X, Y, Z*) coordinates.

Afterwards, a small accumulator array for only the points (causing the ambiguity) located at the peak is constructed using their attributes recomputed from the two new origins. One should note that once the original attributes are computed, re-calculating the attributes from the new origins does not need much computational load. This is expected to lead to the separation of the points causing the ambiguity into different peaks in the accumulator array. Then, plane fitting using the points contributing to the highest peak cell is again conducted. This process is repeated until a good quality of plane fitting is acquired. In the case where the iteration number reaches the maximum (*i.e.*, 5 iterations) without finding a plane fitting result of adequate quality, the next-highest peak cell is detected from the original accumulator array and a plane fitting quality test is carried out.

**Figure 9:**
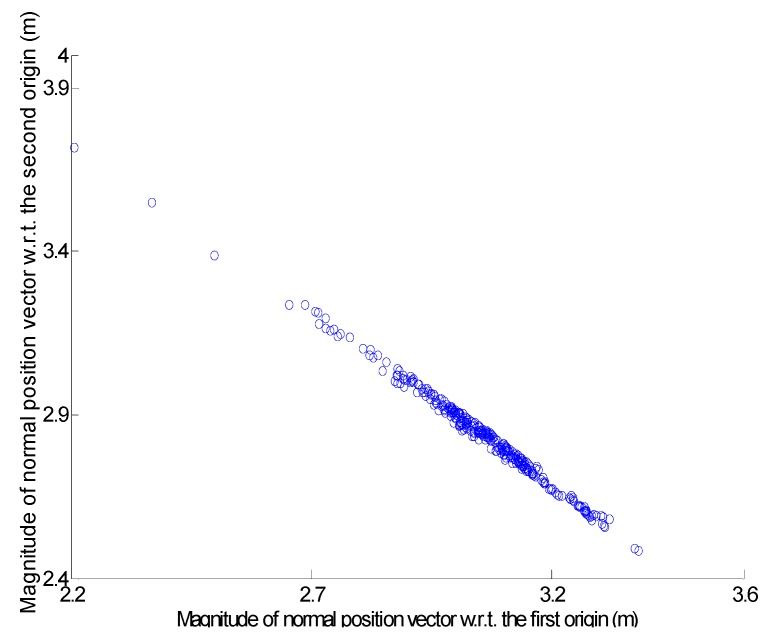
Scatter plot prior to resolution of ambiguity problem.

To verify the performance of the process, two origins were intentionally located at a position having the same XY coordinates (from the center of the gable roof ride line) but different Z coordinates. Figure 9 shows a scatter plot incorporating the magnitudes of the normal position vectors for all of the relevant points on the gable roof. Only one cluster is generated from the points located on the physically different roof patches, due to the intentionally determined locations of the origins. The plane fitting quality measure, which is the RMS of the distances between the points (at the highest peak) and the plane determined, is much worse than the vertical accuracy of the airborne laser scanning data. The plane fitting quality measure for the peak is 0.816 m, which is more than five times the vertical accuracy of the laser data (*i.e.*, 0.15 m). This fact indicates that the peak was constructed by mixed points from two different roof patches.

**Figure 10:**
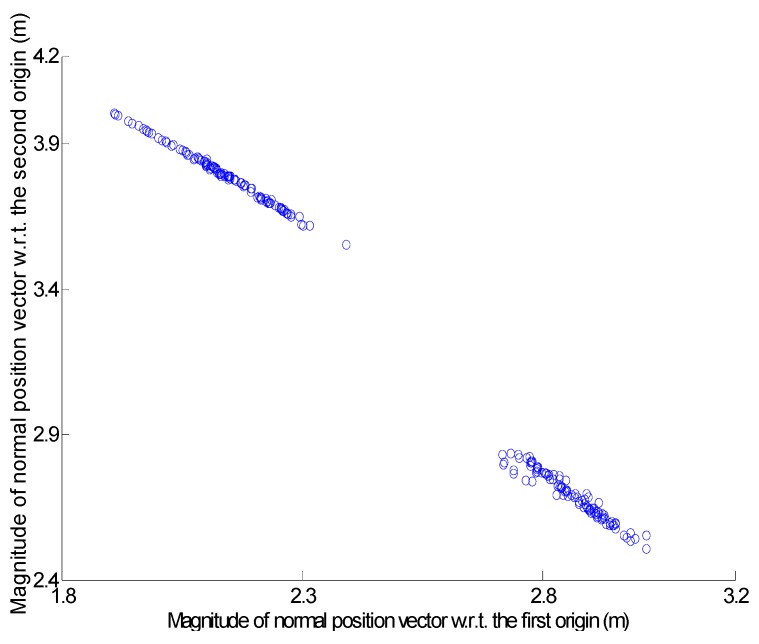
Scatter plot after resolution of ambiguity problem.

The locations of the two new origins were determined by Equation (7). Afterwards, all of the points registered at the peak cell were taken, and their attributes were recomputed using the two new origins. The new accumulator array was constructed based on the recomputed attributes. Figure 10 shows the scatter plot re-drawn using the recomputed attributes of the points registered at the peak cell. There are two distinctively different clusters in the figure. Moreover, the plane fitting quality measure of the highest peak is 0.043 m, which is much better than the vertical accuracy of the airborne laser scanning data. The scatter plot and plane fitting quality measure indicate that the points located on the physically different roof patches produced different clusters.

**Figure 11:**
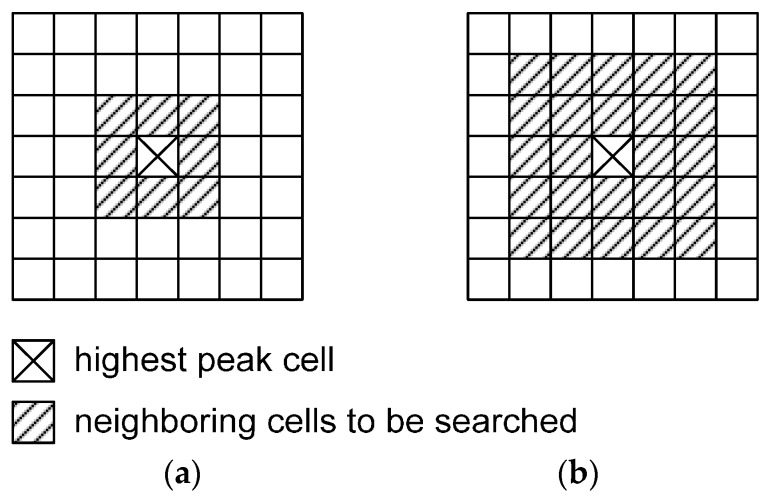
Searching of the neighboring cells in (**a**) the 1st iteration and (**b**) 2nd iteration.

After the homogeneous points registered at the highest peak cell are identified, the remaining clustering steps can be carried out in the original accumulator array based on those points. Then, the clustering process moves to the other points that did not contribute to the highest peak cell but have similar attributes (*i.e.*, the points in the cells neighboring the highest peak). This step is necessary in order to consider the inaccuracy of the computed attributes of certain points, which are generally either located around break lines or affected by noise. More specifically, an initial cluster is first defined by including the points contributing to the highest peak cell. Then, plane parameters are determined by fitting a plane using the points belonging to the initial cluster. Afterwards, the points registered at the cells neighboring the highest peak are identified in the accumulator array. Searching of neighboring cells is carried out by starting from the cells closest to the highest peak and moving thereafter to the more distant ones. As seen in Figure 11a, eight cells neighboring the highest peak cell are searched in the first iteration.

Among the points in these cells, only those that satisfy two conditions, (1) the proximity condition and (2) the co-planarity condition, are included in the initial cluster (refer to Figure 7). More specifically, the points registered at the eight cells are first checked to determine if they are spatially close (*i.e.*, within approximately twice of the average point spacing of the laser data) to the points belonging to the initial cluster (*i.e.*, the proximity condition). Only the points that satisfy the proximity condition are considered as candidates. In other words, the proximity condition is the means of simultaneously checking for the closeness of candidate points in the object domain as well as the similarity of the associated attributes in the parameter domain. Afterwards, the shortest Euclidean distances between the candidate points and the plane defined by the points in the initial cluster are computed. Then, the points that have distances less than the pre-determined threshold are accepted into the cluster (*i.e.*, the co-planarity condition). Here, the threshold is computed based on the noise level of the laser scanning data utilized (e.g., two times the accuracy of the laser data). After this process, the plane parameters are re-determined using all of the points in the cluster. The searching of the neighboring cells in the second iteration is carried out by checking the twenty four neighboring cells, as seen in Figure 11b. The eight neighboring cells that were checked in the first iteration are included in the search areas for the second iteration. Points having similar attributes to the points in the initial cluster but not sufficiently close (*i.e.*, their proximity in the object/spatial domain is not satisfied) to that cluster might still remain in the eight cells; these points are considered in the second iteration. It should be noted that the method of point searching in the neighboring cells is designed to consider such points. Additional points are also included in the cluster after checking the proximity and co-planarity conditions; then, the plane parameters are updated definitively. Afterwards, the iteration proceeds to the next level. This iterative process is continued until no more points can be included in the cluster. Upon completion of this cluster growth, the points in the cluster are recorded and removed from the accumulator array. Now, the second-highest peak cell can be detected, and the same steps implemented for the first-highest peak are repeated. This process of moving from the highest peak to the next highest is repeated until the number of points located at the last peak is less than the number of points that have been pre-determined to make up the minimum detectable patch size in the object space. Figure 8d shows the clustered points categorized into two different groups using the above technique. As it has been mentioned earlier, the clustering process proposed in this research simultaneously considers the similarity in the attribute space and the proximity in the object space. Therefore, the cluster identification and plane parameter evaluation are quite robust since points with higher spatial and attribute similarity take priority over the less homogeneous ones (*i.e.*, with lower degree of similarity and proximity).

## 3. Experimental Results and Evaluations

To analyze the effect of the adaptive cylindrical neighborhood approach on the homogeneity of the laser point attributes, a test with simulated data was carried out in Section 3.1. After then, several experiments using airborne and terrestrial laser data were conducted to verify the performance of the proposed methodology in Section 3.2 to Section 3.3. The relevant issues, including data description, visual inspection of segmentation results, quantitative evaluation, and comparison with other approaches will be addressed sequentially. Additionally, the sensitivity of the thresholds utilized in this study is evaluated in Section 3.4.

### 3.1. Analysis of the Effect of the Adaptive Cylindrical Neighborhood Definition

**Figure 12:**
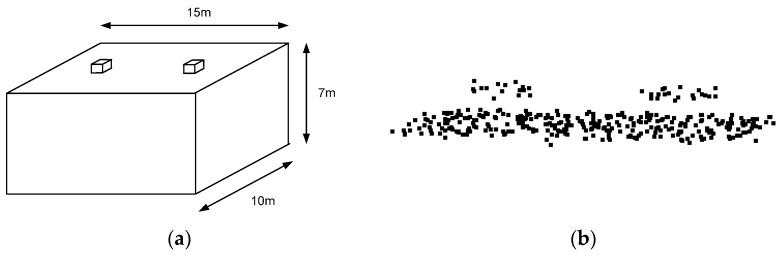
(**a**) Shape of a structure on which laser points are projected; and (**b**) the produced laser points on the rooftop of the structure.

Laser points reflected from a structure of 15 m × 10 m × 7 m dimensions were, first, simulated for this analysis. The structure had two small objects on the rooftop (e.g., cooling towers of 3.0 m × 3.0 m × 1.5 m dimensions). The mean point density of the simulated data was 2.24 points/m^2^ (~0.67 m point spacing), and the horizontal and vertical accuracies of the laser points were 0.5 m and 0.15 m, respectively. It should be emphasized that the simulated data followed the specifications of the real data acquired from OPTECH ALTM 3100, which was used by [32]. Figure 12a,b show the designed structure for the simulation and the produced laser points on the rooftop of the structure, respectively. Three hundred and forty-five (345) laser points were generated, among which thirty-two (32) points were located on the two small objects, as seen in Figure 12b.

**Figure 13:**
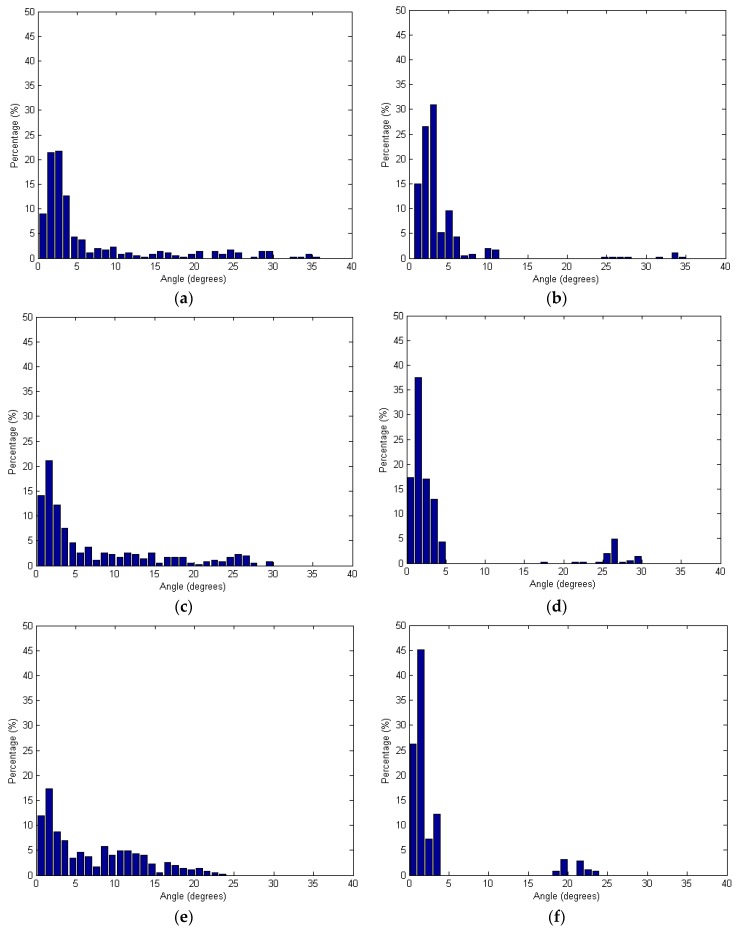

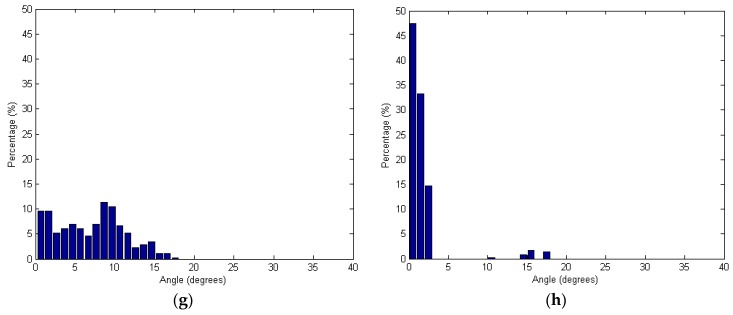
Angles between the surface normal vector calculated and the true one: (**a,c,e,g**) Spherical and (**b,d,f,h**) Adaptive cylindrical approaches applying different radii of 2.0 m (**a,b**); 2.5 m (**c,d**); 3.0 m (**e,f**); and 3.5 m (**g,h**).

Based on the simulated laser points, the fitted plane was determined using the neighboring laser points around the target point; then, the surface normal vector of the plane was calculated. For this test, two neighborhood definition approaches, the adaptive cylindrical and spherical ones, were applied. The surface normal vectors calculated from the two approaches were then compared with the true one. Significantly, the components of the true unit normal vector were (0, 0, 1), because the laser points were reflected from the structure’s horizontal rooftop. Angle computation between the surface normal vector calculated and the true one was implemented for all of the points in the simulated dataset (refer to Equation (8)):

(8) $$\theta_{i}=\text{arccos}(\frac{{\vec{v}}_{i}•{\vec{v}}_{t}}{\left|{\vec{v}}_{i}\right|\left|{\vec{v}}_{t}\right|})$$

where ${\vec{v}}_{i}$ is the surface normal vector of the plane determined using the neighboring laser points around the *i-*th point, ${\vec{v}}_{t}$ is the true unit normal vector (0, 0, 1), and $\theta_{i}$ is the angle between the vectors ${\vec{v}}_{i}$ and ${\vec{v}}_{t}$. In the test, the numbers of neighboring points were determined using four different searching radii: 2.0 m, 2.5 m, 3.0 m, and 3.5 m. Figure 13 shows the angles based on the spherical and adaptive cylindrical approaches together with the searching radii. Additionally, Table 1 lists the statistical evaluation data for the angles calculated.

**Table 1:** Statistical evaluations of the angles between the surface normal vector calculated and the true one.

Radius (m)	Spherical Approach (Degrees)		Adaptive Cylindrical Approach (Degrees)	
	Mean	STD	Mean	STD
2.0	6.738	8.240	4.058	4.991
2.5	7.399	7.872	4.484	7.605
3.0	7.116	5.894	3.257	5.582
3.5	7.018	4.252	1.738	3.067

Figure 13 shows that the angles provided by the adaptive cylindrical approach are more concentrated than the values provided by the spherical one. Considering Table 1 results, it is also clear that the mean and standard deviation values of the angles were much lower for the adaptive cylindrical method than for the spherical one. Therefore, the adaptive cylindrical method can be considered robust against random noises and outliers in the laser point cloud when compared to those using spherical neighborhoods. In other words, the adaptive cylindrical neighborhood approach increases the homogeneity of the laser point attributes. To see the impact of the neighborhood definition on the segmentation results, the qualitative and qualitative comparison between the results from the spherical and adaptive cylindrical approaches is added in Section 3.3, Segments extracted and evaluations.

### 3.2. Real Data Description

Two types of laser scanned data, acquired by airborne and terrestrial laser scanning systems, were utilized for the purposes of the experiments. Three airborne laser scanning datasets were captured over Vaihingen (Germany), Toronto (Canada), and University of Calgary (Canada). One terrestrial laser dataset, additionally, was acquired at Yonsei University (Seoul, Korea). Descriptions of these datasets are summarized in Table 2. The two airborne laser scanning datasets taken over Vaihingen and Toronto have similar point densities. One airborne laser data over University of Calgary has lower point density. On the other hand, the terrestrial dataset has a higher point density than the three airborne datasets.

**Table 2:** Descriptions of Vaihingen, Toronto, and Seoul datasets.

Location	Scanner Type	Airborne/Terrestrial	Point Spacing (m)/Point Density (Points/m^2^)
Vaihingen	Leica ALS50	Airborne	0.38/6.7
Toronto	Optech ALTM-ORION M	Airborne	0.41/6.0
Calgary	Optech ALTM 3100 EA	Airborne	1.15/0.76
Seoul	Leica Scanstation 2	Terrestrial	0.15/44.0

**Figure 14:**
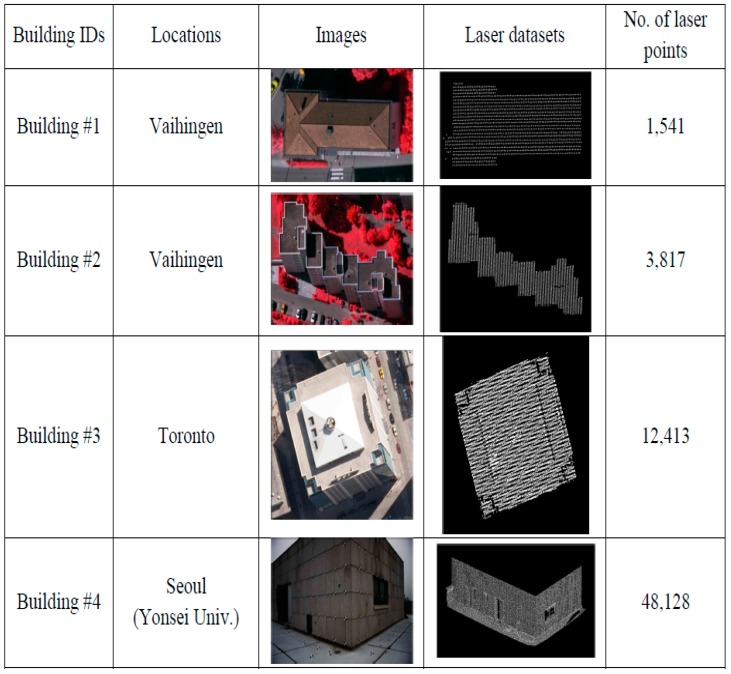
Optical images and displayed laser data of four buildings.

**Figure 15:**
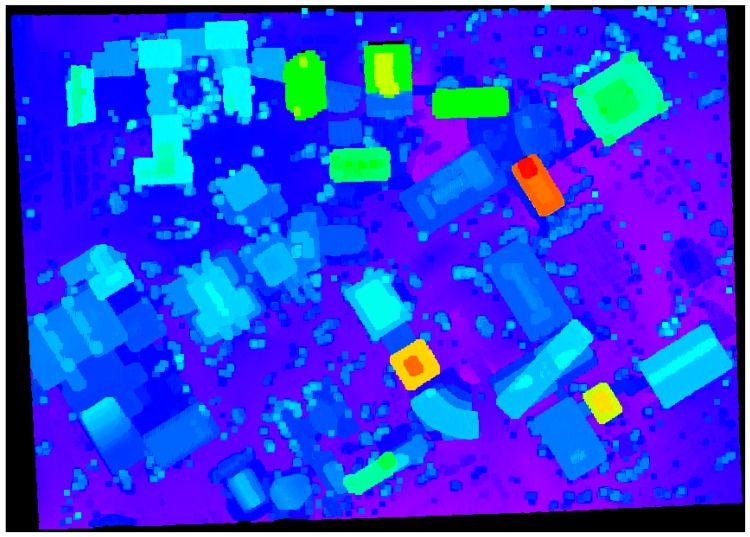
A large dataset over University of Calgary, Canada.

As seen in Figure 14, four buildings from different areas were selected from Vaihingen, Toronto, and Seoul for the experiments. The three buildings in Vaihingen and Toronto included a historic building having a rather complex shape, a high-rise residential building, and a business tower in a modern city, respectively. The forth building, located at Yonsei University, also a modern building, was used as a camera calibration test field. These buildings consisted of planar patches having different slopes, aspects, sizes, heights, and materials. Such buildings are utilized to carry out the detailed quantitative measures (*i.e.*, correctness, completeness, plane fitting precision, plane fitting precision difference, centroid difference, and angle difference values) of the experimental results. Figure 15 illustrates a large dataset taken over University of Calgary, Canada with 320,325 laser points. Also, additional evaluation is carried out using the large dataset over the university.

### 3.3. Segments Extracted and Evaluations

An automated segmentation process based on the proposed methodology was, first, carried out using four different datasets. The flatness of the surfaces, the roughness of the materials, and the instrumental accuracies were considered when the accuracies of the laser datasets in the depth direction were determined. The values from the Vaihingen, Toronto, and Seoul datasets, respectively, were 0.15 m, 0.15 m, and 0.10 m. Descriptions, values, and justifications for the thresholds used in the segmentation process for four buildings are provided in Table 3.

**Table 3:** Descriptions, values, and justifications for the thresholds used in the segmentation process.

Threshold	Description	Value (Building #1/#2/#3/#4)	Justification
Searching radius	Radius of the spherical neighborhood centered at a point	2.00 m/2.00 m/2.00 m/0.30 m	Including sufficient number of points to define reliable attributes
Buffer threshold	Buffer defined above and below the final fitted plane	0.30 m/0.30 m/0.30 m/0.20 m	Two times the accuracy of the laser data in the depth direction
Co-planarity test threshold	Threshold for testing the co-planarity of points at the peak of the accumulator array	0.30 m/0.30 m/0.30 m/0.20 m	Two times the accuracy of the laser data in the depth direction
Proximity threshold	Maximum distance for determining the proximity among points in the object space	0.50 m/0.50 m/0.50 m/0.15 m	Approximately equal to the average point spacing of the laser data
Minimum size of detectable peak	Minimum size of the detected points at the highest peak	4.00 m^2^/4.00 m^2^/4.00 m^2^/0.50 m^2^	Prior knowledge about building planar patch size within the area of interest
Accumulator Cell resolution	Cell size of the accumulator array	0.30 m/0.30 m/0.30 m/0.20 m	Two times the accuracy of the laser data in the depth direction

**Figure 16:**
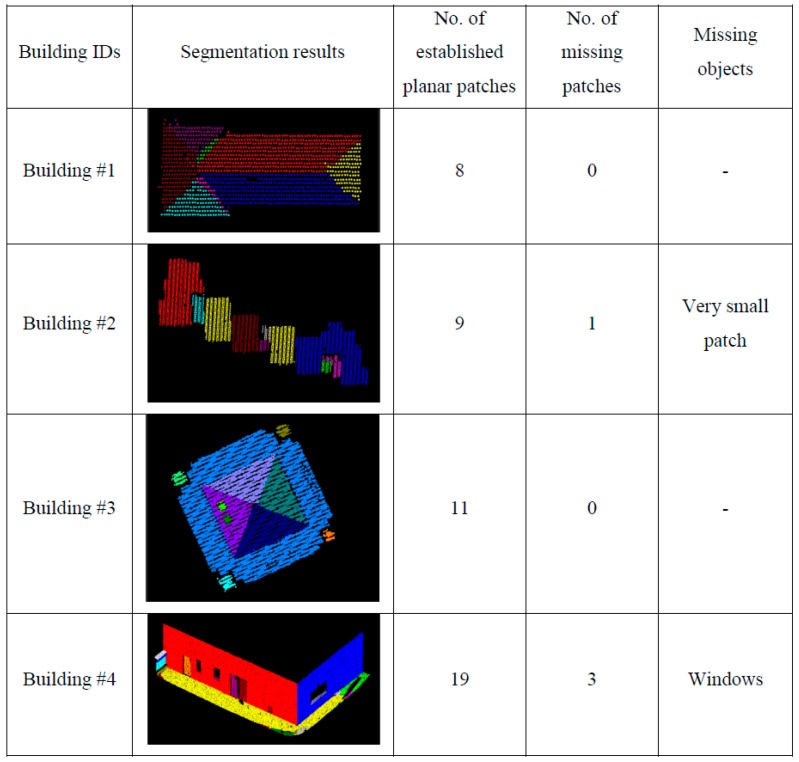
Segmentation results based on the adaptive cylindrical neighborhood definition.

**Figure 17:**
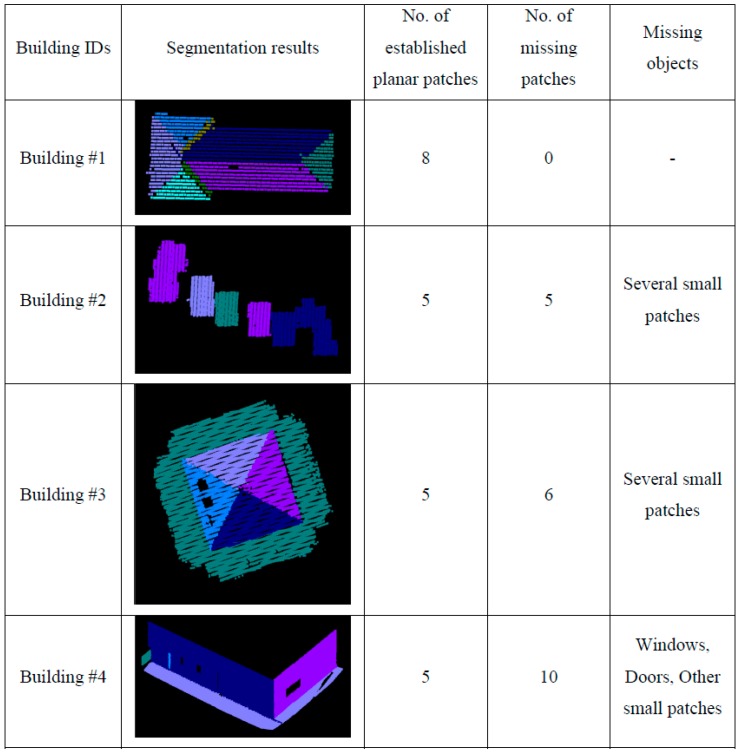
Segmentation results based on the spherical neighborhood definition.

To see the impact of the neighborhood definition on the segmentation results, the adaptive cylindrical and spherical approaches were carried out and compared with each other. More specifically, two different attribute files are generated separately based on the adaptive cylindrical and spherical neighborhood definitions. Afterwards, the exactly same remaining segmentation procedures were applied to those attributes. The final segmentation results based on the adaptive cylindrical and spherical neighborhood definitions are provided in Figure 16 and Figure 17, respectively. Each color indicates a planar patch of different slope and aspect. Figure 16 and Figure 17 also show a number of extracted planar patches, a number of missing patches, and the missing object types. When the segmentation results were visually checked for Figure 16, most of the planar patches had been correctly extracted, excepting a very small object and three windows. The missing objects could be accounted for by the facts that the small patch belonging to building #2 had just very few points and that the windows belonging to building #4 have very noisy and few laser points. On the other hand, the segmentation results based on the spherical neighborhood have many missing objects as seen in Figure 17. Several small patches, windows, and doors are not detected in the segmentation results except for building #1.

For more detailed quantitative evaluations of the experimental results, the automatically extracted patches (*i.e.*, from adaptive cylindrical and spherical approaches) were compared with those manually derived by experienced operators. The manually derived patches functioned in this research as references. Since this research focused on planar segmentation, the laser points belonging to non-planar surfaces were not considered when the manually derived patches were prepared. The correctness, completeness, plane fitting precision, plane fitting precision difference, centroid difference, and angle difference were considered as quantitative measures. The correctness and completeness measures were, first, computed point-by-point, automatic-to-manual comparison of the clustered point IDs. In computing these measures, the total number of laser points with matching IDs, between the automatic and manual clusters, was calculated. The correctness measure in Equation (9) evaluates the percentage of the matched laser points among the established ones (*i.e.*, the automatically detected ones). On the other hand, the completeness measure in Equation (10) provides an indication of the percentage of the matched points among the actual ones (*i.e.*, the manually detected ones). Table 4 and Table 5 list the correctness and completeness values computed from the adaptive cylindrical and spherical approaches for the four buildings, respectively.

(9) $$Correctness=\frac{Total\;number\;of\;laser\;points\;matched}{Total\;number\;of\;laser\;points\;detected\;automatically}$$

(10) $$Completeness=\frac{Total\;number\;of\;laser\;points\;matched}{Total\;number\;of\;laser\;points\;detected\;manually}$$

**Table 4:** Correctness and completeness values of four buildings (Adaptive cylindrical approach).

	No. of Points Detected Manually	No. of Points Detected Automatically	No. of Points Matched	Correctness (%)	Completeness (%)
Building #1	1490	1475	1367	92.68	91.74
Building #2	2988	3007	2946	97.97	98.59
Building #3	11,394	10,509	10,378	98.75	91.08
Building #4	33,778	34,117	32,892	96.41	97.38
Minimum	-	-	-	92.68	91.08
Maximum	-	-	-	98.75	98.59
Overall	49,650	49,108	47,583	96.89	95.84

**Table 5:** Correctness and completeness values of four buildings (Spherical approach).

	No. of Points Detected Manually	No. of Points Detected Automatically	No. of Points Matched	Correctness (%)	Completeness (%)
Building #1	1490	1495	1368	91.51	91.81
Building #2	2988	2632	2624	99.70	87.82
Building #3	11,394	10,305	10,133	98.33	88.93
Building #4	33,778	35,660	31,097	87.20	92.06
Minimum	-	-	-	87.20	87.82
Maximum	-	-	-	99.70	92.06
Overall	49,650	50,092	45,222	90.43	91.08

In case of adaptive cylindrical approach (as seen in Table 3), the minimum and maximum values of the correctness measure were 92.68%, and 98.75%, respectively. The minimum and maximum values of the completeness measure were 91.08%, and 98.59%, respectively. In terms of the overall correctness and completeness measures, the adaptive cylindrical segmentation algorithm provided 96.89% and 95.84% values, respectively. On the other hand, the spherical approach (as seen in Table 4) showed worse segmentation results compared to the adaptive cylindrical one. In this case, the minimum and maximum values of the correctness measure were 87.20%, and 99.70%, respectively. The minimum and maximum values of the completeness measure were 87.82%, and 92.06%, respectively. In terms of the overall correctness and completeness measures, the spherical segmentation algorithm provided 90.43% and 91.08% values, respectively. Those values mostly affected by the results from buildings #2 and #4. As seen in Table 4, building #2 has much less number of points detected automatically than the points detected manually. This caused the maximum value of the correctness (*i.e.*, 99.70%) and the minimum value of the completeness (*i.e.*, 87.82%). However, it is the other way around in case of building #4. There are much more automatically detected points than the manually detected ones. This caused the minimum value of the correctness (*i.e.*, 87.20%) and the maximum value of the completeness (*i.e.*, 92.06%).

After the segmentation process completed, plane fitting was carried out for all of the automatically and manually derived planar patches. In this process, the plane fitting precisions are determined by calculating the RMS errors of the planes. Also, the centroids of the fitted planes and angles of the normal position vectors are calculated. Afterwards, the differences between the automatically derived planes and the corresponding manual ones are computed in terms of the plane fitting precisions, the centroids of the fitted planes, and the angles of the normal position vectors. For the case of the adaptive cylindrical approach, Table 6, Table 7, Table 8 and Table 9 show the values of these measures for buildings #1 to #4, respectively. Table 10, Table 11, Table 12 and Table 13 also show the evaluation results for the spherical approach. It needs to be noted that the bottom of building #4, because it is not planar, was not considered in the evaluation process. As Figure 16 illustrates, the bottom of the building consists of several patches, not a single patch.

**Table 6:** Plane fitting precision, plane fitting precision difference, centroid difference, and angle difference values for building #1 (Adaptive cylindrical approach).

Patch	Precision-Manual (m)	Precision-Auto (m)	Precision Difference (m)	Centroid Difference (m)	Angle Difference (Degree)
P1	0.025	0.051	0.026	0.304	0.685
P2	0.027	0.030	0.003	0.381	0.052
P3	0.032	0.033	0.002	0.140	0.173
P4	0.032	0.031	−0.001	0.133	0.086
P5	0.026	0.029	0.003	0.149	0.100
P6	0.022	0.022	0.000	0.130	0.064
P7	0.037	0.020	−0.017	0.675	0.347
P8	0.031	0.016	−0.014	1.298	1.588
Mean	0.029	0.029	0.000	0.401	0.387

**Table 7:** Plane fitting precision, plane fitting precision difference, centroid difference, and angle difference values for building #2 (Adaptive cylindrical approach).

Patch	Precision-Manual (m)	Precision-Auto (m)	Precision Difference (m)	Centroid Difference (m)	Angle Difference (Degree)
P1	0.020	0.027	0.007	0.017	0.020
P2	0.023	0.032	0.009	0.025	0.016
P3	0.052	0.053	0.001	0.015	0.002
P4	0.019	0.037	0.019	0.038	0.069
P5	0.016	0.068	0.052	0.169	1.973
P6	0.016	0.031	0.015	0.196	0.847
P7	0.021	0.012	−0.009	0.415	0.285
P8	0.017	0.046	0.028	0.148	1.575
P9	0.015	0.073	0.058	0.885	7.901
Mean	0.022	0.042	0.020	0.212	1.410

**Table 8:** Plane fitting precision, plane fitting precision difference, centroid difference, and angle difference values for building #3 (Adaptive cylindrical approach).

Patch	Precision-Manual (m)	Precision-Auto (m)	Precision Difference (m)	Centroid Difference (m)	Angle Difference (Degree)
P1	0.027	0.049	0.022	0.072	0.086
P2	0.034	0.035	0.002	0.227	0.029
P3	0.035	0.042	0.007	0.436	0.025
P4	0.025	0.024	−0.001	0.136	0.017
P5	0.115	0.086	−0.029	0.280	0.003
P6	0.095	0.091	−0.004	0.145	0.906
P7	0.070	0.074	0.005	0.131	1.233
P8	0.094	0.046	−0.048	0.039	1.471
P9	0.101	0.085	−0.016	0.265	1.530
P10	0.021	0.020	−0.001	0.629	0.040
P11	0.080	0.044	−0.036	1.267	7.960
Mean	0.063	0.054	−0.009	0.330	1.209

**Table 9:** Plane fitting precision, plane fitting precision difference, centroid difference, and angle difference values for building #4 (Adaptive cylindrical approach).

Patch	Precision-Manual (m)	Precision-Auto (m)	Precision Difference (m)	Centroid Difference (m)	Angle Difference (Degree)
P1	0.005	0.006	0.002	0.053	0.017
P2	0.002	0.002	0.000	0.066	0.001
P3	0.009	0.007	−0.002	0.371	0.135
P4	0.001	0.001	0.000	0.015	0.020
P5	0.004	0.002	−0.002	0.063	0.196
P6	0.002	0.002	0.000	0.018	0.034
P7	0.002	0.001	0.000	0.008	0.031
P8	0.002	0.002	0.001	0.017	0.398
P9	0.001	0.005	0.004	0.123	2.477
P10	0.001	0.007	0.006	0.035	3.365
Mean	0.003	0.004	0.001	0.077	0.667

**Table 10:** Plane fitting precision, plane fitting precision difference, centroid difference, and angle difference values for building #1 (Spherical approach).

Patch	Precision-Manual (m)	Precision-Auto (m)	Precision Difference (m)	Centroid Difference (m)	Angle Difference (Degree)
P1	0.025	0.049	0.024	0.366	0.690
P2	0.027	0.027	0.000	0.358	0.021
P3	0.032	0.034	0.003	0.047	0.122
P4	0.032	0.029	−0.002	0.107	0.085
P5	0.026	0.018	−0.008	0.182	0.168
P6	0.022	0.041	0.019	0.541	0.676
P7	0.037	0.031	−0.006	0.220	2.225
P8	0.031	0.027	−0.004	0.578	2.594
Mean	0.029	0.032	0.003	0.300	0.823

**Table 11:** Plane fitting precision, plane fitting precision difference, centroid difference, and angle difference values for building #2 (Spherical approach).

Patch	Precision-Manual (m)	Precision-Auto (m)	Precision Difference (m)	Centroid Difference (m)	Angle Difference (Degree)
P1	0.020	0.022	0.002	0.182	0.026
P2	0.023	0.019	−0.004	0.014	0.008
P3	0.052	0.052	0.000	0.024	0.007
P4	0.019	0.019	0.001	0.021	0.020
P5	0.016	N.A.	N.A.	N.A.	N.A.
P6	0.016	N.A.	N.A.	N.A.	N.A.
P7	0.021	N.A.	N.A.	N.A.	N.A.
P8	0.017	N.A.	N.A.	N.A.	N.A.
P9	0.015	N.A.	N.A.	N.A.	N.A.
Mean	0.022	N.A.	N.A.	N.A.	N.A.

**Table 12:** Plane fitting precision, plane fitting precision difference, centroid difference, and angle difference values for building #3 (Spherical approach).

Patch	Precision-Manual (m)	Precision-Auto (m)	Precision Difference (m)	Centroid Difference (m)	Angle Difference (Degree)
P1	0.027	0.057	0.030	0.221	0.105
P2	0.034	0.044	0.010	0.476	0.103
P3	0.035	0.032	−0.003	0.586	0.040
P4	0.025	0.022	−0.004	0.186	0.031
P5	0.115	0.094	−0.022	0.210	0.017
P6	0.095	N.A.	N.A.	N.A.	N.A.
P7	0.070	N.A.	N.A.	N.A.	N.A.
P8	0.094	N.A.	N.A.	N.A.	N.A.
P9	0.101	N.A.	N.A.	N.A.	N.A.
P10	0.021	N.A.	N.A.	N.A.	N.A.
P11	0.080	N.A.	N.A.	N.A.	N.A.
Mean	0.063	N.A.	N.A.	N.A.	N.A.

**Table 13:** Plane fitting precision, plane fitting precision difference, centroid difference, and angle difference values for building #4 (Spherical approach).

Patch	Precision-Manual (m)	Precision-Auto (m)	Precision Difference (m)	Centroid Difference (m)	Angle Difference (Degree)
P1	0.005	0.027	0.022	0.098	0.179
P2	0.002	0.004	0.002	0.104	0.011
P3	0.009	N.A.	N.A.	N.A.	N.A.
P4	0.001	0.001	0.000	0.023	0.008
P5	0.004	N.A.	N.A.	N.A.	N.A.
P6	0.002	0.011	0.009	0.205	4.072
P7	0.002	N.A.	N.A.	N.A.	N.A.
P8	0.002	N.A.	N.A.	N.A.	N.A.
P9	0.001	N.A.	N.A.	N.A.	N.A.
P10	0.001	N.A.	N.A.	N.A.	N.A.
Mean	0.003	N.A.	N.A.	N.A.	N.A.

Upon close examination of Table 6, Table 7, Table 8 and Table 9 for the adaptive cylindrical approach, it is evident that building #2 has the maximum value of mean precision difference (*i.e.*, around 0.02 m) compared with the other three buildings. As for the centroid difference, the mean value for building #1 (*i.e.*, around 0.4 m) is greater than those of the other buildings. In the case of the angle difference, buildings #2 and #3 show around 1.4 and 1.2 degrees, respectively. Among the four buildings’ 38 patches, P8 in building #1, P9 in building #2, and P11 in building #3 significantly affected the values of three measures: precision difference, centroid difference, and angle difference. The numbers of laser points belonging to the three patches (the manually derived ones) were quite small (*i.e.*, 40, 42, and 84, respectively). It should be emphasized that the plane fitting quality of patches having a small number of points can be more affected by missing points than is the case for patches having a large number of points. On the other hand, building #4 showed quite good values for the three measures.

In case of Table 10, Table 11, Table 12 and Table 13 for the spherical approach, there are many missing patches (*i.e.*, which are expressed as N.A.) except for building #1. The three measures, which are plane fitting precision, precision difference, and centroid difference for building #1 in Table 10, are similar to the values from the adaptive cylindrical one in Table 6. However, the angle difference between the approaches is a bit different due to P7 and P8. Building #2 in Table 11 shows a slightly better result than that in Table 7. It is also evident that the evaluation results for buildings #3 and #4 in Table 12 and Table 13 are worse than those in Table 8 and Table 9.

Additionally, the mean values of the centroid and angle differences were computed, as listed in Table 14, using all of the patches (*i.e.*, 38 patches) belonging to the four buildings, so as to confirm the overall performance of the adaptive cylindrical segmentation algorithm. At this stage, one should note that the results from the spherical approach are not considered for the overall mean values of the centroid and angle differences since it produced many missing patches. It is also important to note that neither the plane fitting precision nor the plane fitting precision difference are included in Table 14, since the characteristics of the laser points (*i.e.*, the degree of laser point accuracy) in building #4 differ so markedly from those in the other three datasets. Overall, the mean value of centroid difference was 0.25 m, and the angle difference was less than 1°, as can be seen in Table 14.

**Table 14:** Overall mean values of centroid difference and angle difference measures (Adaptive cylindrical approach).

	Centroid Difference (m)	Angle Difference (Degree)
Overall mean	0.250	0.941

**Figure 18:**
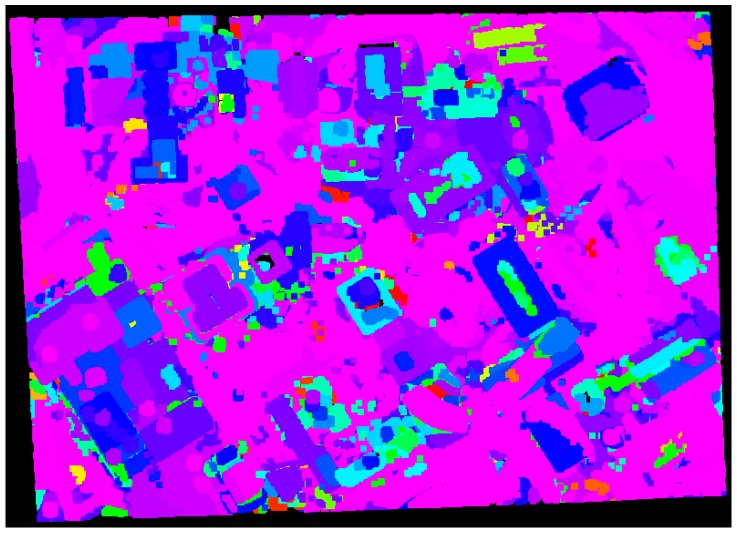
Segmentation results based on the region growing approach over University of Calgary.

**Figure 19:**
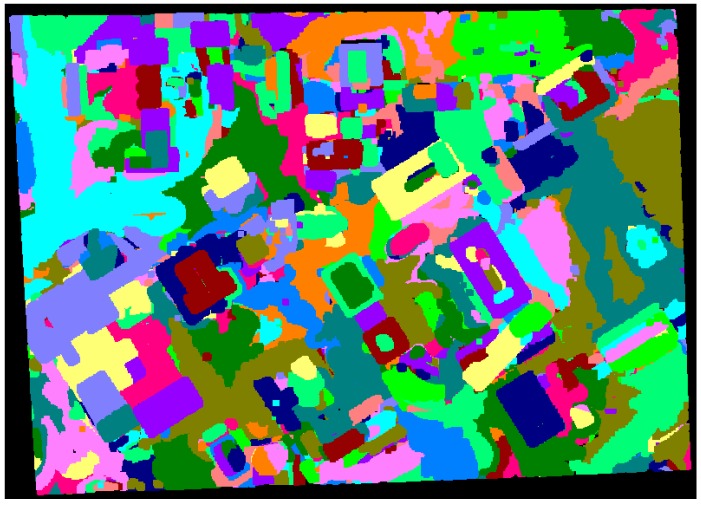
Segmentation results based on the proposed approach over University of Calgary.

Additional experiment using the proposed approach (*i.e.*, the adaptive cylindrical approach) with a large dataset taken over University of Calgary, Canada (refer to Figure 15) was carried out while comparing the result from the region growing approach [4,5,6,7,8,28], which is well-known as a segmentation algorithm. Figure 18 and Figure 19 illustrate the segmentation results from the region growing and the proposed approach, respectively. As seen in Figure 18, the segmentation procedure based on the region growing approach produced 2718 different patches. Moreover, many circular patches can be detected in the figure. Such result shows that the region growing approach causes the significant over-segmentation and there is sensitivity to the choice of seed points. On the other hand, the proposed approach produced a better segmentation results (with 1499 patches) compared to the region growing one as illustrated in Figure 19. A few patches which are over-segmented are found in the figure; however, most ones are segmented correctly. The ones with irregular shapes are planar patches extracted from the terrain or roads.

**Figure 20:**
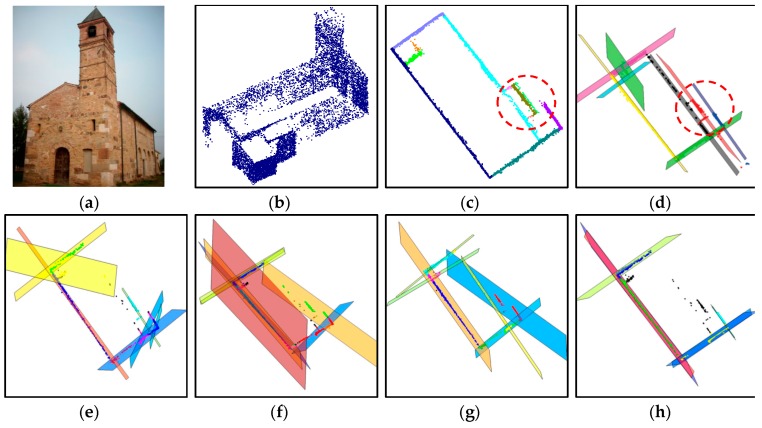
Result comparison using Pozzoveggiani dataset: (**a**) image of Pozzoveggiani church; (**b**) 3D points of Pozzoveggiani church; (**c**) top view of the result from the proposed approach; (**d**) result from J-linkage; (**e**) result from Residual Histogram Analysis (RHA); (**f**) result from MultiRANSAC; (**g**) result from sequential RANdom SAmple Consensus (RANSAC); and (**h**) result from mean-shift.

The segmentation performance of the proposed approach is also compared with J-linkage, RHA, multiRANSAC, sequential RANSAC, and mean-shift. The comparisons are carried out by using the same dataset as [14] (*i.e.*, a 3D point cloud data of Pozzoveggiani church, Italy; see Figure 20a,b). One should note that the 3D point data of the church is actually derived from a set of images. The 3D plane extraction results using the above-mentioned algorithms are taken from [14] and shown in Figure 20d–h. As seen in Figure 20c, the proposed approach derived 11 planar surface segments. J-linkage also has 10 planar surfaces as seen in Figure 20d. Except for the proposed approach and J-linkage, the other approaches extract the surfaces incorrectly or have missing ones as seen in the figures.

**Figure 21:**
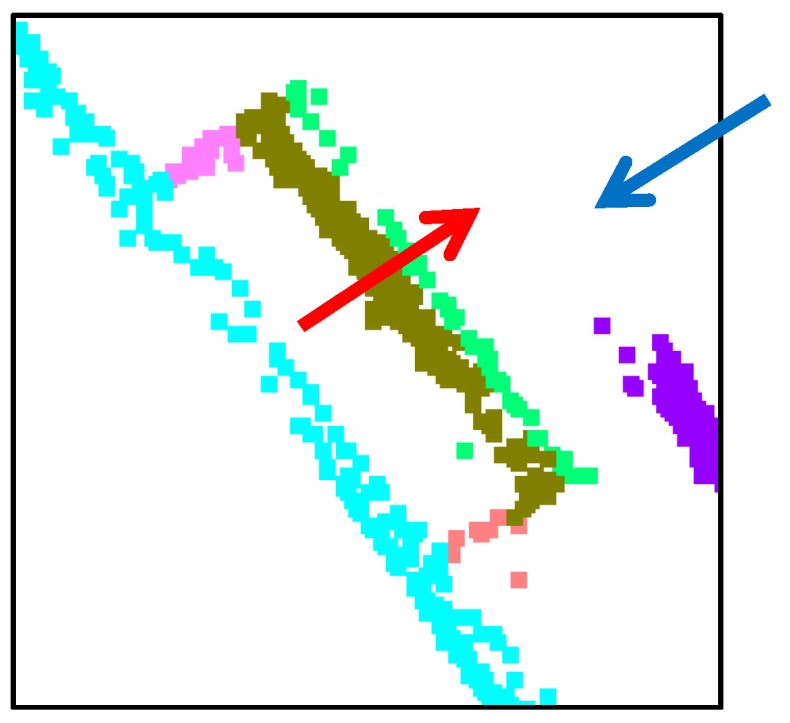
Enlarged area which is enclosed by the red dashed circles in Figure 20c.

The only difference between the proposed approach and J-linkage is on the areas enclosed by the red dashed circles in Figure 20c–d. To take a closer look at the area in Figure 20c, it is enlarged as seen in Figure 21. Two segments indicated by the red and blue arrows are separated and derived using the proposed approach; however, J-linkage does not have two separated planes. The result comparison shows that the proposed approach works well with the 3D point data derived even from a set of images. Moreover, the result from the proposed approach is a bit better than the J-linkage and superior to the other approaches (*i.e.*, RHA, MultiRANSAC, Sequential RANSAC, and mean- shift).

### 3.4. Evaluation of the Sensitivity of the Thresholds

The proposed approach uses several thresholds in the segmentation process such as (1) searching radius; (2) buffer threshold; (3) co-planarity test threshold; (4) proximity threshold; (5) minimum size of detectable peak; and (6) accumulator cell resolution (please refer to the justifications of these thresholds in Table 3). First of all, *proximity threshold* is defined as the value which is approximately equal to the average point spacing of the laser data. The average point spacing of the laser data is, usually, provided by a data vendor or can be simply calculated. The other threshold, *minimum size of detectable peak*, is defined as the value which is based on prior knowledge about building planar patch size within the area of interest. This threshold can be changed according to the minimal planar patch size that the user wants to derive for his or her purpose of work. Hence, at this stage, the authors can mention that the two thresholds, *proximity threshold* and *minimum size of detectable peak* are not significant values and can be determined easily.

The other three thresholds, which are *buffer threshold*, *co-planarity test threshold*, and *accumulator cell resolution* are all defined as the same value which is two times the accuracy of the laser data in the depth direction (refer to the justification in Table 3). Hence, knowing the accuracy of the laser data is significantly important to determine these three thresholds. Similar to the average point spacing of the laser data, the accuracy of the laser data is usually known or provided by a data vendor. For example, as addressed in Section 3.3, the values from the Vaihingen, Toronto, and Seoul datasets, respectively, were given as 0.15 m, 0.15 m, and 0.10 m. On the other hand, the remaining threshold, *searching radius*, is defined as the value which is for including sufficient number of points to define reliable attributes. Even though the values of the four thresholds (*i.e.*, buffer threshold, co-planarity test threshold, accumulator cell resolution, and searching radius) can be determined properly and easily as above-mentioned, the additional analyses are carried out in this section to evaluate the sensitivity of the thresholds while changing the values of the accuracy of the laser data and searching radius.

**Figure 22:**
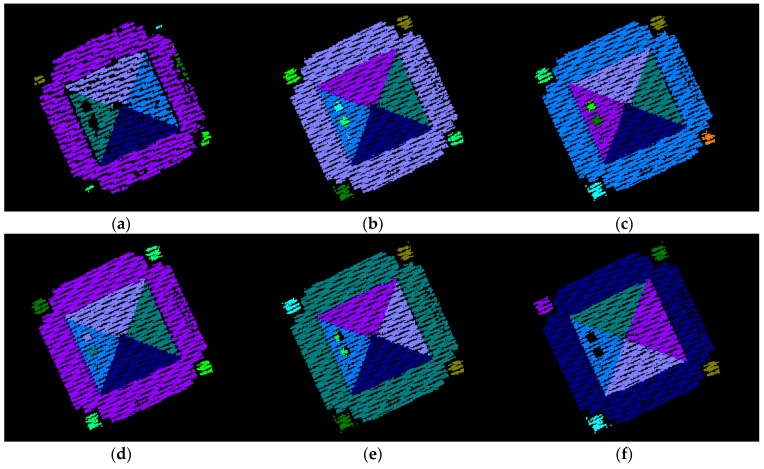
Segmentation results according to changing the threshold, searching radius, from 0.5 m to 5.0 m (the accuracy of the laser data is 0.15 m for all cases). Searching radius: (**a**) 0.5 m; (**b**) 1.0 m; (**c**) 2.0 m; (**d**) 3.0 m; (**e**) 4.0 m; (**f**) 5.0 m.

For this analysis, building #3 in Toronto data (see Figure 14) is utilized. The first analysis is about the threshold, searching radius while changing the values from 0.5 m to 5 m. When radius is 0.5 m, the segmentation process missed several patches located at the four corners and the left roof side of the building as can be seen in Figure 22a. Moreover, the segmented patches do have less number of points compared to the other cases (*i.e.*, the cases of Radius of 1.0 m to 5.0 m). This comes from the small searching radius which causes unreliable attribute computation due to the insufficient number of points for neighborhood definition. On the other hand, the good segmentation results are acquired and they are almost similar to each other when the radius is increased (*i.e.*, 1.0 m to 3.0 m, also see Figure 22b–d). However, in case of too large searching radius (more than 4.0 m), the results were losing two small patches at the left roof side of the building (Figure 22e–f). Also, large searching radius increases the computational load since the number of points in the neighborhood is too large. In summary, searching radius can be determined to include sufficient number of points to define reliable attributes. Moreover, the thresholds from 1.0 m to 3.0 m derived good segmentation results; which means that the range of the proper searching radius threshold is wide enough in the proposed approach.

**Figure 23:**
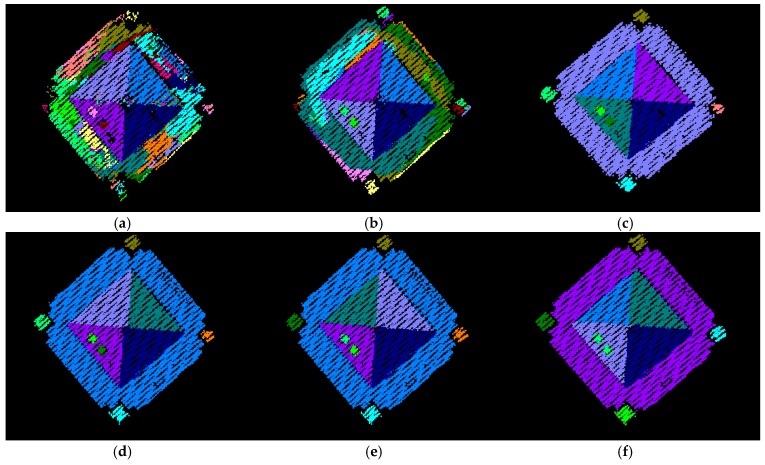
Segmentation results according to changing the accuracy of the laser data from 0.02 m to 0.30 m (searching radius is 2.0 m for all cases). The accuracy of the laser data: (**a**) 0.02 m; (**b**) 0.05 m; (**c**) 0.10 m; (**d**) 0.15 m; (**e**) 0.20 m; (**f**) 0.30 m.

The second analysis is about the accuracy of the laser data which determines the three thresholds such as buffer threshold, co-planarity test threshold, and accumulator cell resolution. Recall that the accuracy of the laser data is usually known or provided by a data vendor (0.15 m for this dataset). However, the segmentations are carried out for this analysis while intentionally changing the accuracy values in the process from 0.02 m to 0.3 m. When the laser data accuracy is set to 0.02 m, over-segmentation occurs; hence, lots of planar patches are acquired from the segmentation process (in Figure 23a). Similar but less segments are acquired for the case of the value of 0.05 m (Figure 23b). The phenomenon of the over-segmentation is reduced when the value applied is increased. On the other hand, almost same segmentation results are acquired when the values of 0.10 m to 0.30 m are applied (Figure 23c–f). With a closer look at the segmentation results in these figures, the size of the small planar patch at the right corner of the building is slightly increasing when the value of the applied accuracy is increasing. This means that it will be fine to apply the value of the accuracy from 0.10 m to 0.30 m. In other words, the range of the proper value of the laser data accuracy can be from little bit less than to greater than the value provided (*i.e.*, 0.15 m for this dataset). However, one should note that the large value of the applied laser data accuracy causes more computational load since the number of points in the neighborhood will increases. Hence, to avoid the over-segmentation and keep the computational efficiency, the value of the laser data accuracy can be set close to the one provided.

## 4. Conclusions and Research Recommendations

Ever since the importance of laser scanning measurement was first recognized, various laser point classification and segmentation approaches have been proposed. However, prior segmentation techniques suffer from limitations such as sensitivity to the choice of seed points, lack of consideration of the spatial relationships among points, and inefficient performance. The segmentation approach newly proposed in this paper provides four main benefits while circumventing the addressed limitations of the existing techniques.

(1) Neighborhood definition, which considers both the three-dimensional relationships between random points and the shapes of surfaces, is introduced; this increases the homogeneity among neighbors, which is crucial to the achievement of reliable point attributes. By means of a test using simulated data, it was established and verified that the homogeneity level from the adaptive cylindrical method is much better than that from the spherical one. Also, the high level of attribute homogeneity leads to the better segmentation results.

(2) A voting scheme with a 2D accumulator array was introduced to aggregate points having similar attributes into clusters. In this clustering approach, the magnitudes of normal position vectors are utilized as new laser point attributes. Methods for determining the locations of the origins and avoiding the segmentation ambiguity are suggested in this research. The computational efficiency in terms of memory requirement is enhanced, accordingly, by reducing the dimensions of the utilized accumulator array.

(3) The clustering procedure proposed in this research simultaneously considers the similarity in the attribute space and the proximity in the object space. Furthermore, points with a higher level of homogeneity take priority over those with lower levels. These two operations in the process of updating plane parameters and defining point members belonging to clusters allow the proposed segmentation approach to provide a fairly robust solution.

(4) The methodology proposed works well with both airborne and terrestrial laser point clouds. The results of experiments using real airborne and terrestrial data demonstrate, through qualitative and quantitative evaluations, the outcomes’ high level of reliability. The proposed segmentation algorithm, moreover, provides quite high overall correctness and completeness values (*i.e.*, 96.89% and 95.84%, respectively). Furthermore, the overall mean value of centroid difference was 0.25 m, and the angle difference was less than 1°. The performance of the proposed approach was also verified with a large dataset. In the comparison with other approaches, the proposed one showed the better result than others.

In conclusion, the methodology proposed herein is a robust and efficient means of abstracting an enormous number of laser points into plane information. Upcoming research will turn its attention more toward origin-location determination methods that do not cause any segmentation ambiguity and accumulator array dimension reduction by using only one origin. Additionally, the proposed methodology’s focus on planar patches will be extended to deal with non-planar surfaces of spherical or cylindrical shape. This research will also focus on the automated neighborhood size determination.
